# Photocatalytic Activity and Self-Cleaning Effect of Coating Mortars with TiO_2_ Added: Practical Cases in Warm Sub-Humid Climates

**DOI:** 10.3390/ma17010190

**Published:** 2023-12-29

**Authors:** Liliana Kuk-Dzul, Luis F. Jiménez, Ricardo E. Vega-Azamar, Mayra P. Gurrola, Julio C. Cruz, Danna L. Trejo-Arroyo

**Affiliations:** 1 Tecnológico Nacional de México/I.T. de Chetumal, Av. Insurgentes 330, Chetumal 77013, Mexico; clilianakd@gmail.com (L.K.-D.); luis.jt@chetumal.tecnm.mx (L.F.J.); ricardo.va@chetumal.tecnm.mx (R.E.V.-A.); 2 IxM-CONAHCYT-Tecnológico Nacional de México/I.T. de Chetumal, Av. Insurgentes 330, Chetumal 77013, Mexico; mayra.pg@chetumal.tecnm.mx

**Keywords:** photocatalytic activity, cement-based coating mortar, limestone aggregates

## Abstract

In this study, the photocatalytic activity of coating mortars with synthetized and commercial TiO_2_ nanoparticles added has been evaluated at 2, 3 and 5% by weight of cement by calculating the degradation efficiency of methyl orange and red wine dyes exposed to both visible-light and UV radiation; also, the self-cleaning effect of coatings exposed to weather conditions (warm sub-humid climate) was assessed. TiO_2_ nanoparticles were synthesized via the sol–gel method to a low synthesis temperature and characterized via X-ray diffraction (XRD) and Scanning Electron Microscopy (SEM). The results show synthesized TiO_2_ particles in anatase phase with a crystallite size of 14.69 nm, and hemispherical particles with sizes of submicron order. The addition percentage with the best performance in the coating mortars was 3%, with both commercial and synthesized TiO_2_; however, coating mortars with synthesized TiO_2_ exhibited the highest degradation efficiency for both dyes when they were exposed to visible light, while mortars with commercial TiO_2_ exhibited the highest degradation efficiency when exposed to UV radiation. In addition, in coating mortars with synthesized TiO_2_, the self-cleaning effect was evident from the beginning of exposure to weather, reaching the largest dye-free surface at the end of exposure. The compressive strength increased significantly in mortars with TiO_2_ addition.

## 1. Introduction

The biodeterioration of cementitious surfaces exposed to weather is one of the main problems for any infrastructure throughout its useful life, and is more prominent in warm sub-humid climates. This biodeterioration can be accelerated depending on the climatic conditions in the area to which it is exposed [1]. Cement-based coating materials applied to infrastructure surfaces are often exposed to varying environmental conditions, such as solar radiation at different intensities (depending on latitude and season), increased atmospheric pollution in urban areas [2], local climatic conditions (wind, rain, temperatures) and severe environmental loads (as in coastal zones), causing a gradual deterioration in the surface properties, which can modify the structural characteristics in buildings, bridges, roads, pavements and historical monuments, among others, generating high maintenance costs and environmental impact [3]. In addition, cement-based materials such as mortars and concrete, in any kind of structures, are the ideal substrates for the growth of fungal micro-organisms, algae, lichens, etc., due to their microporous structure [4,5,6], thus attacking any surface exposed to weather [7], especially in locations near to the coast with tropical climates and highly saline environments, hot temperatures and high relative humidity, which, apart from being unaesthetic, can also generate health issues. 

Coating mortar, as a cement-based material, is one of the most important construction materials and is used most frequently in all civil works as the surface envelope in different types of buildings. An integral part of coating mortar is the fine aggregate used. Regarding this, the fine aggregate of limestone origin has a content of about 73% calcium carbonate (CaCO_3_); additionally, this aggregate has an irregular-type surface and a rather rough texture [8], and has a high percentage of porosity and a high percentage of water absorption. Water in limestone constructions is one of the main causes of biodeterioration, since it penetrates into the pores causing different physical phenomena. Furthermore, water can carry organic and inorganic components that can dissolve the mineral matrix. Physicochemical environmental factors generated under the conditions of high temperatures, high relative humidity, saline environments, sunlight and wind plus the surface defects generated on the surfaces of coating mortars such as pores and roughness, among others, give rise to the inoculation and growth of micro-organisms in the cementitious surface [9]. Currently, new trends in cement systems are being developed, such as nanoscale modification [10], to increase the performance of cement-based materials and provide them with functional characteristics with the incorporation of photocatalytic materials that activate their potential to solve energy problems and pollution remediation [11]. 

Regarding photocatalytic materials, they are activated with UV radiation from the sun or other light source to degrade organic, inorganic and microbial pollutants adsorbed on surfaces or present in the air [9,12,13]. Likewise, extensive research on photocatalytic materials has been focused on wastewater treatment of organic contaminants derived from different industries [14,15], such as the removal of antibiotics from the pharmaceutical industry [16] and the petroleum industry [17], among others. In recent decades, one of the most studied photocatalytic materials has been titanium dioxide (TiO_2_) [18] in the form of anatase [19,20,21] due to its low process requirements, low cost, non-toxicity and its ability to degrade an extensive range of organic contaminants. On the other hand, TiO_2_ is added to cementitious materials (cement paste, mortars and concrete) as it exhibits photocatalytic properties to produce self-cleaning and air purification applications [22,23]. In the construction industry, commercial TiO_2_ is already used for photocatalytic applications such as paints and cement-based composites; it has been used to generate surfaces that are self-cleaning on exposure to light through photocatalytic destruction of organic materials. However, the photocatalytic efficiency and self-cleaning effect in cementitious coatings with limestone aggregates must be evaluated in real environmental conditions and even able to improve the durability of cement-based materials exposed to weather, considering the nature of the substrate and certain environmental conditions. Concerning photocatalytic efficiency, colloidal suspensions of TiO_2_ have been applied as coatings on limestone rocks with different surface characteristics (roughness, porosity and cohesion). The literature has stressed that the stability of the coatings applied on large surfaces is directly related to the substrate characteristics, together with a poor dispersion of TiO_2_, since the adherence to the surface and the photocatalytic efficiency can be affected [24]. On the other hand, it has also been reported that photocatalytic efficiency is not always related to surface roughness, and it is more appropriate to evaluate the available active surface [25]. In addition, it has been identified that a surface with porosity and roughness has an advantage in the retention of TiO_2_ particles. It is important to highlight the need for additional studies to develop and integrate photocatalytic materials for building construction, which are practical, efficient and low-cost. Evaluating that the influence of different percentages of TiO_2_ addition on the photocatalytic action, particle sizes, and the way whichnwhichch it is incorporated in the manufacturing process of cementitious coatings remains a topic of debate [18].

This work researches the photocatalytic performance of coating mortars with limestone aggregates and the addition of anatase-TiO_2_ nanoparticles at a low synthesis temperature, based on the degradation of methyl orange and red wine dyes as a model for the destruction of organic contaminants on surface coating mortars exposed to visible light and UV radiation. The self-cleaning effect of coating mortars exposed to weather for two months was also evaluated. The environmental conditions corresponded to tropical (subhumid warm) weather with both high temperature and relative humidity, and a saline environment characteristic of coastal areas.

## 2. Materials and Methods

### 2.1. TiO_2_ Nanoparticles Synthesis and Characterization

Titanium dioxide (TiO_2_) nanoparticle synthesis was performed through the sol–gel method [26,27], from the precursor titanium isopropoxide (IV) (Sigma-Aldrich, St. Louis, MO, USA, 97%). Shortly, absolute ethanol (Merck KGaA, Darmstadt, Germany EMSURE, 99.7%) was used as solvent; 28.6% of the titanium isopropoxide precursor was added to 71.4% of ethanol and left under constant stirring for 30 min; and pH < 2 was controlled with hydrochloric acid (HCl) (J.T. Baker, Phillipsburg, NJ, USA, 36.5–38.0%) until a gel was obtained, which was placed in a drying oven and kept at 80 °C for 24 h. The solid material was calcined at a temperature of 400 °C for 1 h. Physicochemical characterization of both synthesized and commercial TiO_2_ (RA, Fermont) powders was performed by means of Scanning Electronic Microscopy (SEM) with a JEOL JSM-6010PLUS/LA microscope, equipped with an X-ray Dispersive Energy Spectroscope (EDS). For the identification of the phases, an X-ray diffraction analysis (XRD) was performed on a Ray Diffractometer Bruker D8 Advanced Davinchi with a copper Kα line radiation (CuKα) and, from the peak intensity of the DXR pattern, crystallite size was determined by means of the Debye–Scherrer formula [28]. 

### 2.2. Mortar Mixtures 

The materials used were made with white ordinary Portland cement and a limestone fine aggregate passing sieve 16 (1.18 mm), from crushed limestone obtained in a local market, previously characterized in [8]. Methyl orange and red wine were used as colorants and are considered organic pollutants; they were chosen as examples of polluting substances and their degradation was taken as indication of photocatalytic and self-cleaning ability. Physical characterization of the aggregates was carried out by means of a granulometry test, according to [29]. Mortar mixtures were designed with a cement/fine aggregate ratio of 1:3 and a water/cement ratio (w/c) of 0.6. Synthesized TiO_2_ nanoparticles were added at 2, 3 and 5% by weight of cement and, for comparative purposes, mortar samples with addition of commercial TiO_2_, at the same concentrations, were also prepared. A readjustment of the w/c ratio to 0.7 for the mortar mixtures with TiO_2_ additions was based on the flow tests as a control parameter (110 ± 5%) [30]. The TiO_2_ nanoparticles were previously dispersed dry in an agate mortar, subsequently dispersed in distilled water on a magnetic grill with constant stirring for 30 min, and the final dispersion was carried out in an ultrasonic tub for 25 min. Finally, they were added to the cementitious matrix as mixing water. Mortar mixtures were cast in cylindrical plastic molds (Ø6 × 1.5 cm). They were left to set for 24 h in a black box and subsequently cured in water for 28 days at room temperature. In total, 36 mortar samples with TiO_2_ additions (18 corresponding to synthesized and the same amount to commercial TiO_2_; 6 samples for each concentration in both cases) and 6 samples of mortar without additions were tested. These mortars without TiO_2_ additions were designated as reference mortars RM and mortar samples with addition of synthesized TiO_2_ were designated as TSM, and different concentrations of 2, 3 and 5% by weight of cement were designated as TSM2, TSM3 and TSM5, respectively. As for the mortar samples with additions of commercial TiO_2_ nanoparticles, they were designated as TCM2, TCM3 and TCM5, respectively.

### 2.3. Exposure to the Weather Conditions 

Coating mortar samples RM, TSM and TCM with the different addition percentages of both synthesized and commercial TiO_2_ were immersed in prepared solutions of red wine (red wine/water ratio of 1:1) and methyl orange (1 × 10^−3^ mg/100 mL) (Technical Chemistry Inc., Cranston, RI, USA) for 24 h for total saturation. Subsequently, they were placed on a plate, to be exposed to the weather for two months. Monitoring was carried out every 2 weeks and recorded with digital photography to visually analyze the self-cleaning effect in mortars from the degradation of dyes. It should be noted that the environmental loads of the mortars exposed to the weather were those corresponding to coastal areas with saline environments and high temperatures and relative humidity for most of the year.

### 2.4. Photocatalytic Tests

The photocatalytic behavior of RM and TSM and TCM mortar samples, exposed to both UV irradiation and visible light, was determined by monitoring the degradation in the concentration of red wine and methyl orange. After 28 days of curing, the mortar samples were removed from water and allowed to dry in the black box at room temperature for 48 h. The analysis was performed using a UV-Vis spectrophotometer (Genesys, Menlo Park, CA, USA, 10uv). The samples were irradiated with UV (Illumination lamp, LUMIACTION, Taipei, Taiwan, Lumiaction lamp, model PL9/UV 1H 9W UV, spectrum used for the two lamps was 395 nm) for 30, 60, 90, 120, 150, 180, 210, 240 and 1440 min (24 h), as well as exposed to visible light for the same time. The distance between the UV lamp and the samples was 35 cm. The concentration of the dyes was measured using the main absorption peak control at the wavelength of 510 nm. The photocatalytic activity of the mortar samples was calculated through the removal efficiency [31,32] using the following equation (Equation (1)):

*A* (%) = [(*Co* − *C*)/*Co*] × 100, (1)

where *Co* is the concentration of red wine and methyl orange in the dark at time = 0 min and *C* is the concentration of the exposed sample exposed at different measurement times.

### 2.5. Compressive Strenght Test

Based on the results obtained for the photocatalytic activity behavior, the compressive strength test was carried out only on the preselected RM, TSM3 and TCM3 mortars. This mechanical resistance test was performed as described in [33], which consisted of determining the maximum compressive strength that the material resists through the gradual application of an axial load in two opposite faces of the specimens’ square section until failure occurs. The test was carried out in a hydraulic press.

## 3. Results and Discussion

### 3.1. TiO_2_ Nanoparticle Characterization

**Figure 1:**
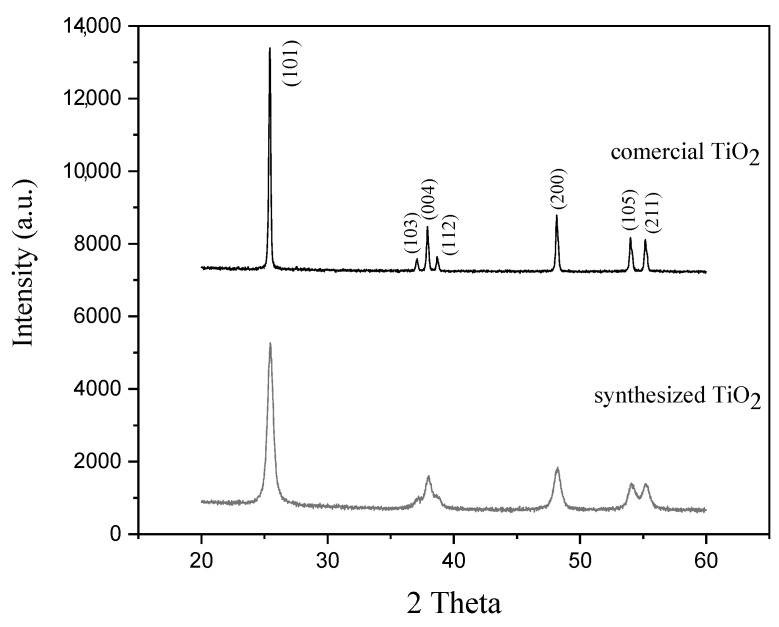
XRD pattern of commercial and synthesized TiO_2_ powders calcined at 400 °C for 1 h.

The graph in Figure 1 shows the X-ray diffraction pattern of both synthesized calcined TiO_2_ nanoparticles at 400 °C for 1 h and commercial TiO_2_ nanoparticles. As a result of the indexation of the peaks, only peaks corresponding to the anatase phase of titanium dioxide were found, and no characteristic peaks corresponding to another phase such as brookite were observed that could be generated as a result of the conditions of the use of an acid medium for precipitation [19]. TiO_2_ synthesized in the laboratory showed that the peaks of anatase phase partly appeared at 2θ = 25.388° (101), 37.083° (103), 37.946° (004), 38.597° (112), 48.134° (200), 53.976° (105) and 55.077° (211). The indexation of the peaks of commercial TiO_2_ corresponded to the anatase phase (PDF No.73-1764). Based on the XRD pattern, the material formed has a tetragonal crystal structure with lattice parameters a = 3.776 Å and c = 9.486 Å. It is important to mention that with the increase in temperature, around 550 °C, the transformation to the rutile phase is achieved [21,24,34], which is not the objective of this work. The mean crystallite size was determined using the D. Scherrer formula, taking the full width at half the maximum peak values and using the wavelength value of 0.15418. The mean crystallite sizes of the anatase phase, calculated from the (101) diffraction peak of the synthesized TiO_2_ and the commercial one, were found to be 14.69 and 36.43 nm, respectively; thus, the values obtained for the synthesized TiO_2_ powders were much smaller than those for the commercial ones. The synthesized TiO_2_ powders were used under these conditions for the preparation of subsequent coating mortar samples and the evaluation of their photocatalytic activity.

**Figure 2:**
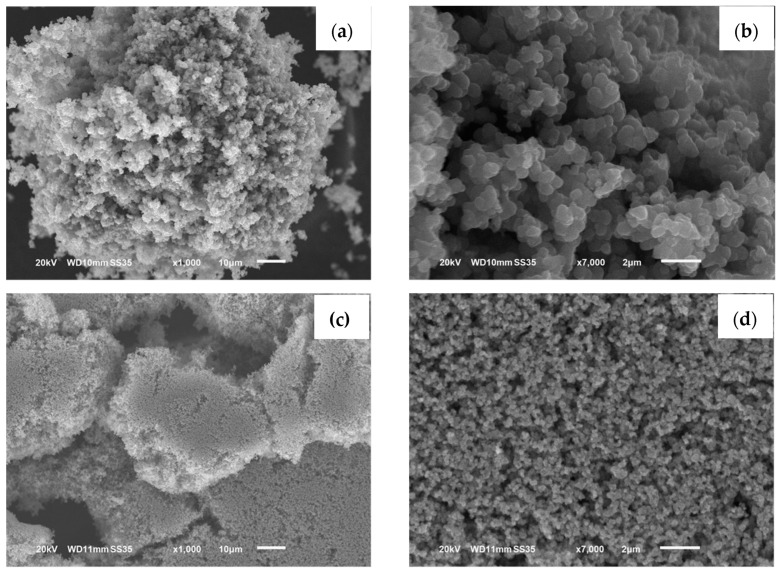
SEM micrographs of TiO_2_ powders: (**a**) synthesized TiO_2_, 1000×, (**b**) synthesized TiO_2_, 7000×, (**c**) commercial TiO_2_, 1000×, (**d**) commercial TiO_2_, 7000×.

The micrographs obtained via SEM for both the synthesized and commercial TiO_2_ powders are presented in Figure 2. The image in Figure 2a corresponds to the micrograph of the synthesized TiO_2_ powders at 1000× magnification, in which clusters of diminutives particles with narrow particle size distribution of submicron order can be observed. The image in Figure 2b corresponds to the same powders at 7000× magnification, in which the semi-spherical morphology of the particles can be appreciated in greater detail; the average particle size estimated from the micrograph was ~0.59 µm, with narrow particle size distribution, which also presented a higher degree of agglomeration. It is worth mentioning that at this stage the synthesized TiO_2_ powders had not been subjected to any dispersion or grinding process, they were analyzed directly from how they were obtained after synthesis. The image in Figure 2c corresponds to a micrograph of the commercial TiO_2_ powders at 1000× magnification, in which clusters of particles much smaller than those of the synthesized TiO_2_ powders can be observed. At 7000× magnification (Figure 2d), commercial TiO_2_ powders with a homogeneous dispersion of semi-spherical morphology particles can be seen in greater detail, and the estimated average particle size was 0.2 µm. However, the crystallite size of the synthesized TiO_2_ was approximately 50% smaller than that of the commercial TiO_2_, suggesting a larger surface area that can result in more active sites in the photocatalyst.

### 3.2. Photocatalytic Activity in Coating Mortars

**Figure 3:**
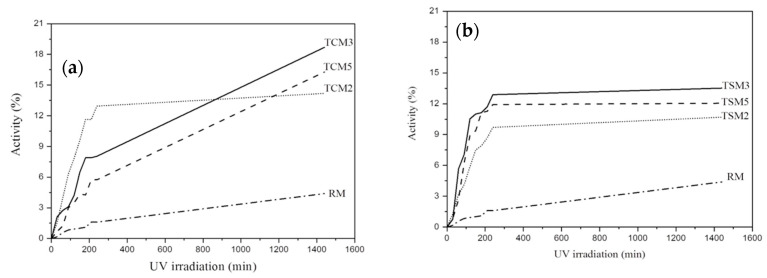
Photocatalytic activity in coating mortar samples exposed to red wine and irradiated with UV: (**a**) TCM mortars, (**b**) TSM mortars.

Figure 3 depicts the photocatalytic activity of coating mortars with the addition of both commercial and synthesized TiO_2_ nanoparticles, exposed to the red wine solution and irradiated with UV. Figure 3a corresponds to the mortars added with commercial TiO_2_ (TCM) and the reference mortar (RM). TCM3 mortars showed the highest photocatalytic activity, with degradation efficiency for the red wine above 18% after 24 h. The TCM5 mortar, despite the higher TiO_2_ content, showed a lower photocatalytic activity and degradation efficiency, around 16%. TCM3 and TCM5 presented a linear increase behavior; their degradation efficiency was gradually increased within the UV irradiation time interval, which could indicate an additional increase in photocatalytic activity over time. For the case of TCM2 mortars, the photocatalytic activity gradually increased during the first 3.5 h; from there, no increase over time was observed, and a steady state was reached, with a degradation efficiency of 14%. As for RM, it presented very low dye removal efficiency, reaching only 4% after 24 h.

Regarding the coating mortar samples with synthesized TiO_2_ additions (Figure 3b), the TSM3 mortars also showed the highest photocatalytic activity, and also had the highest degradation efficiency of 13.5%. In descending order, the TSM5 mortars showed a degradation efficiency of 12% and the TSM2 mortars of approximately 10.6%. All TSM mortars presented a gradual increase until 3.5 h of UV irradiation; subsequently, a steady state was reached for the determined time interval, suggesting that there is no further increase in photocatalytic activity for longer irradiation times. The photocatalytic activity of both TCM and TSM was greater for coating mortars with 3% TiO_2_ addition, followed by mortars with 5% added, mortars with 2% and finally the reference mortars.

**Figure 4:**
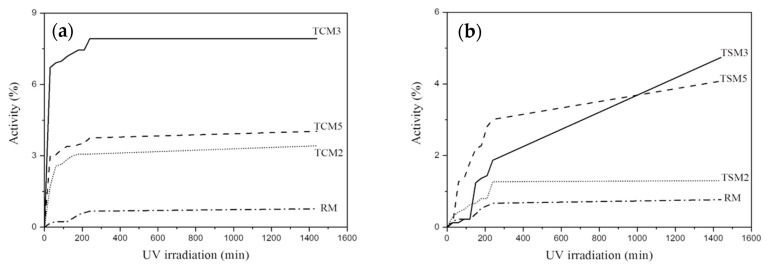
Photocatalytic activity in coating mortar samples exposed to methyl range irradiated with UV. (**a**) TCM mortars, (**b**) TSM mortars.

The photocatalytic activity of the TCM and TSM mortars, exposed to methyl orange and irradiated with UV, is presented in Figure 4. The same trend may be observed for the above-discussed mortars. However, in this case, the maximum value of the photocatalytic activity reached a degradation efficiency of 8.8% for the TCM3 mortar (Figure 4a). As for the previous mortars, in descending order, the photocatalytic activity of the TCM5 mortar occupied the second place with a degradation efficiency of 4.5%, the TCM2 mortars had a degradation efficiency of 3.6% and, finally, for RM an efficiency below 1% was obtained. The curve behavior in Figure 4a indicates that the methyl orange degradation efficiency for all TCM mortars reached a steady state after 4 h of UV irradiation. It was found that there is a greater removal efficiency of red wine than of methyl orange dye in TCM mortars exposed to UV irradiation. In Figure 4b, the photocatalytic activity of the TSM mortars is presented. Again, TSM3 presented the highest photocatalytic activity with a degradation efficiency of 4.7%, while TSM5 presented only 4%; both the TSM3 and TSM5 samples have an upward slope, which suggests an increase in activity over time. TSM2’s degradation efficiency was 3.4%, and these mortars reached a stationary state behavior after 2.5 h of UV irradiation. Coating mortars with addition of synthesized TiO_2_ showed a lower degradation efficiency for both red wine and methyl orange dyes than mortars with commercial TiO_2_ additions when exposed to UV radiation.

**Figure 5:**
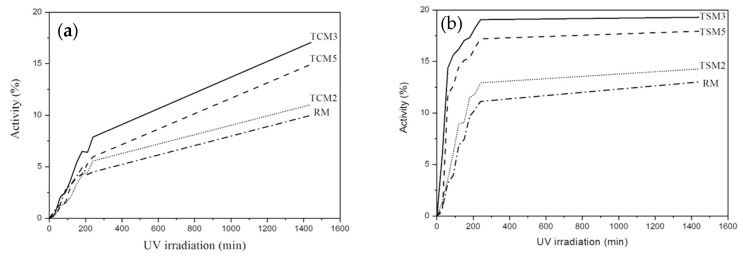
Photocatalytic activity of coating mortar samples exposed to red wine and irradiated with visible light: (**a**) TCM mortars, (**b**) TSM mortars.

In the graphs of Figure 5, the data obtained from the photocatalytic activity of mortars exposed to red wine and irradiated with visible light are presented. Figure 5a corresponds to TCM mortars. Once more, TCM3 presented the highest photocatalytic activity with a degradation efficiency of 17%, while the TCM5 and TCM2 mortars showed a degradation efficiency of 15 and 11%, respectively. In all specimens, a linear increase was observed in the studied time interval, which suggests an additional increase in photocatalytic activity over time. Unlike the TCM mortars, the TSM mortars presented a higher photocatalytic activity, with higher degradation rates for red wine when exposed to visible light. In this case, TSM3 reached a degradation efficiency of 19.3%, TSM5 reached 18% and, finally, TSM2 reached 14.3% (Figure 5b). It should be noted that a gradual increase was observed until 3.5 h of exposure and, from there, a steady-state behavior was noticed for the 24 h time interval.

**Figure 6:**
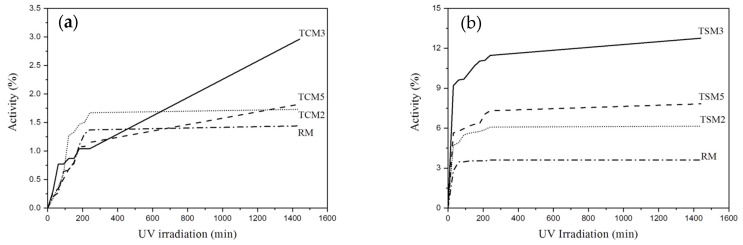
Photocatalytic activity of coating mortar samples exposed to methyl orange and irradiated with visible light: (**a**) TCM mortars, (**b**) TSM mortars.

The degradation results for methyl orange for mortars exposed to visible light are presented in Figure 6. The analysis of these results produced similar trends in terms of the order of response of the TCM and TSM mortars. However, the TSM mortars (Figure 6b) presented the highest photocatalytic activity with degradation efficiencies for methyl orange of 12.7%, 7.5% and 6% (TSM3 > TSM5 > TSM2), respectively. Only mortars with 3% of TiO_2_ showed an increase after the exposure time of 24 h; the rest of the mortars presented a steady state after 3.5 h. While the TCM3 mortars reached only 3% photocatalytic activity after 24 h of exposure, the TSM3 mortars reached 12.7% under the same conditions.

The difference in photocatalytic activation between the coating mortars with synthesized and commercial TiO_2_ is evident when they are exposed to visible light and UV irradiation. The coating mortars with the addition of synthesized TiO_2_ presented greater photocatalytic activity percentages when exposed to visible light, while the mortars with the addition of commercial TiO_2_ presented greater photocatalytic activity when they were exposed to UV irradiation. In the case of TiO_2_ synthesized at 400 °C, an anatase phase was obtained, as well as a particle size of the submicron order, semi-spherical morphology and a crystallite size of 14.69 nm, 50% smaller compared to commercial TiO_2_, which gives rise to a larger surface area that generates greater absorption capacity and more active sites for photocatalysis [9]. Photocatalysis is induced when a semiconductor, in this case TiO_2_, is irradiated with an energy source greater than its band gap energy (band gap is 3.2 eV for anatase, 380 nm wavelength). The semiconductor absorbs a photon, causing electron–hole pairs to be generated. These electron–hole pairs act as oxidizing or reducing agents when they encounter surface groups or molecules absorbed on their surface [13]. The coating mortars with the addition of synthesized TiO_2_ nanoparticles absorbed visible light (>380 nm) and exhibited a substantial enhancement in visible-light-assisted photocatalytic degradation of red wine and methyl orange. This suggests that synthetized TiO_2_ nanoparticles have a lower band gap that the commercial TiO_2_ nanoparticles, since less energy was required to induce photocatalysis when they were exposed to visible light [35]. These results represent two great practical benefits: the first is that TiO_2_ nanoparticles are synthetized at low calcination temperatures (400 °C), which minimizes the energy consumption required for them be obtained, and this agrees with [20]; and the second is that mortars with these synthesized TiO_2_ (TSM) nanoparticles present greater photocatalytic activity when exposed to visible light, noting that visible light contains 43% of the energy of the global solar irradiation spectrum of the air mass. The rest comes in the form of near-infrared radiation (52%) or UV radiation (5%). Therefore, their photoactive capacity increases when they are exposed to longer wavelengths.

### 3.3. Degradation of Dyes in Coating Mortars Exposed to the Weather Conditions

**Figure 7:**
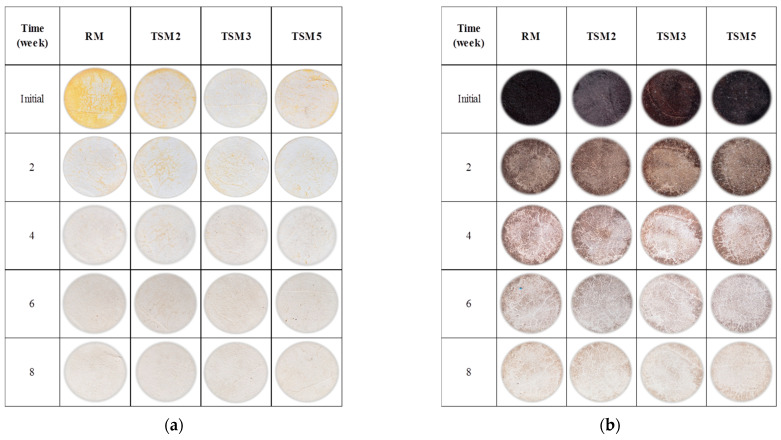
Photographs of coating TSM mortars contaminated and exposed to the weather for two months: (**a**) methyl orange, (**b**) red wine.

In the image of Figure 7, the results for the RM and TSM mortars are presented qualitatively (digital photographs). After the immersion process for 24 h in the dye solutions, methyl orange (Figure 7a) and red wine (Figure 7b), both types of coating mortar were exposed to the weather for two months. In general, red wine presented a greater degree of dirtiness on the surface, which indicates that red wine favors the proliferation of micro-organisms such as mold [27], a phenomenon that was not the focus of this investigation. In the initial week, the TSM mortars with all TiO_2_ additions showed a lower degree of impregnation of the solutions than the RM mortars, which was attributed to a lower porosity achieved by the coating mortars due to the effect of the TiO_2_ nanoparticles and which was reflected in the increase in mechanical resistance shown in the results of the compression resistance tests. Nevertheless, TSM3 (for methyl orange) and TSM2 (for red wine) presented a lower degree of dirtiness on their exposed surface. Progressively, after 8 weeks, all mortars were effectively self-cleaned, presenting a clearer surface with fewer imperfections caused by the contaminant. 

**Figure 8:**
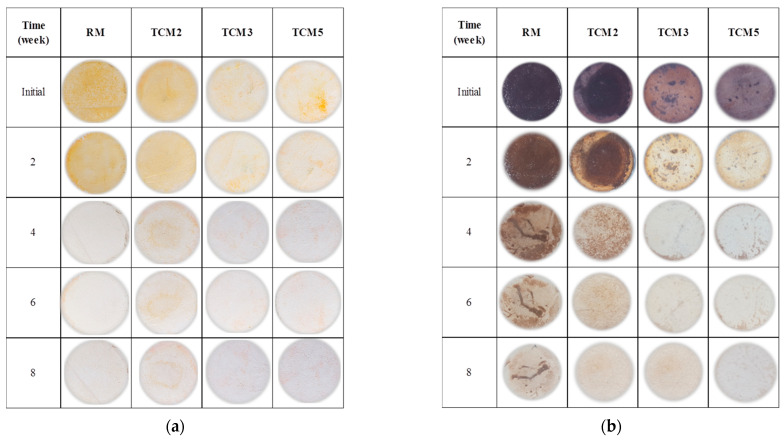
Photographs of coating TCM mortars contaminated and exposed to the weather for two months: (**a**) methyl orange, (**b**) red wine.

In Figure 8’s image, the progress in the self-cleaning process in the TCM mortars submerged in the methyl orange solution (Figure 8a) and in red wine (Figure 8b) is shown, both after 24 h immersion and after being exposed to the weather for two months. On the exposed surfaces of the mortars, once again, it can again be observed that, in the initial week, the mortars with TiO_2_ presented a lower degree of dirtiness from both dyes when compared to RM. In addition, the TCM3 mortar had the lowest degree of dirtiness for both dyes. The self-cleaning effect was progressively observed in all mortars, showing greater color degradation as the exposure time increased. The qualitative comparative analysis between the TSM and TCM mortars indicated that TSM degraded the red wine more than the methyl orange dye, while TCM degraded the methyl orange more effectively. 

On the other hand, during the preparation of the coating mortars, the synthesized and commercial TiO_2_ nanoparticles were initially dispersed in water, and this water with the nanoparticles was used as the mixing water. Therefore, part of this water was absorbed directly by the limestone aggregates (considered to have high absorption) [8,36], suggesting that some nanoparticles may be distributed directly in the limestone aggregates, and due to their hydrophilic nature and photocatalytic activity they can react with the water molecules or organic components [18,37,38]. When oxidant radicals (•OH) are generated, a series of chemical reactions are triggered, which cause the bonds of the dye molecules to separate until the entire contaminant is mineralized; oxygen radicals (•O^2−^) also contribute to the elimination of contaminants [15]. In this way, they can reduce the nucleation and proliferation of contaminants or micro-organisms inside the mortar.

However, the TSM3 and TCM3 mortars presented a greater self-cleaning effect in all cases, which agrees with the results presented previously in terms of the greater photocatalytic activity in the mortars with the 3% addition of TiO_2_ nanoparticles. The coloration in the initial week of the mortars was not constant; however, it is not a function of the increase in TiO_2_ content, and the effect is attributed to a greater absorption of dyes during the impregnation process. 

**Figure 9:**
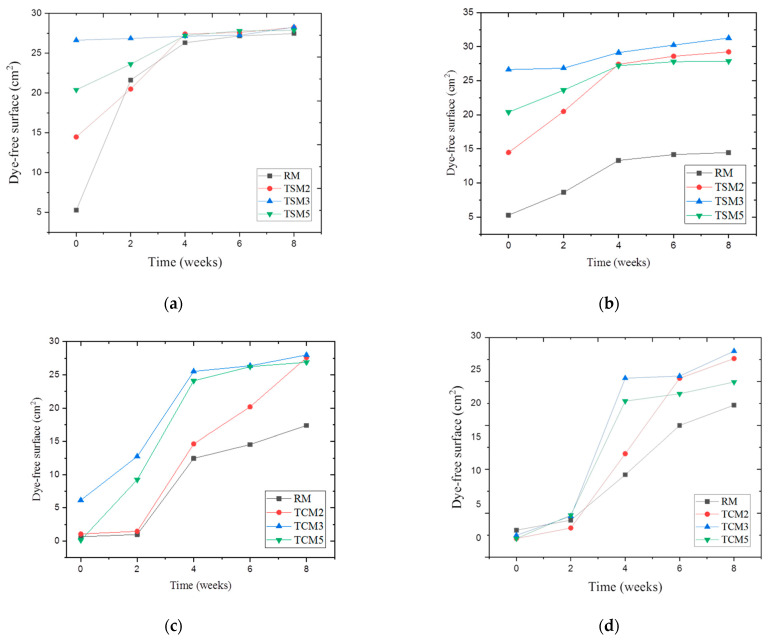
Semi-quantitative analysis of the self-cleaning effect in coating mortars exposed to the weather conditions for two months: (**a**) TSM mortars (methyl orange), (**b**) TSM mortars (red wine), (**c**) TCM mortars (methyl orange), (**d**) TCM mortars (red wine).

In a semi-quantitative manner, the self-cleaning effect on the exposed surface of the coating mortars was calculated by means of ImageJ version 1.51j8 image processing software. The results are presented in the graphs of Figure 9. After immersion of the mortars in the dyes for 24 h, the initial surface coloration was recorded. 

The RM reference mortars showed the highest absorption of dyes, with a stained surface area of 94.8% for those submerged in methyl orange and 100% for red wine. The self-cleaning effect in the coating mortars as a function of the dye-free surface area is presented in the graphs of Figure 9. Figure 9a,b correspond to the TSM mortars in the methyl orange and red wine solutions, respectively, where the TSM3 mortar shows a self-cleaning effect from the beginning of exposure, and also the highest clean surface values. Furthermore, they achieved a greater self-cleaning effect when they were exposed to red wine. It is highlighted that the rest of the mortars (TSM2 and TSM5) showed a better performance from the second week of exposure, stabilizing in the fourth week. In terms of the TCM mortars in the methyl orange solution (Figure 9c) and in red wine (Figure 9d), it is shown that, for all mortars, the self-cleaning effect appears after the third week, with the best performance for TCM3 and the poorest for RM, with all stabilizing between the sixth and eighth week, with the best performances as follows: TCM3, TCM2, TCM5 and RM, in decreasing order. For all cases, after eight weeks of exposure to the weather conditions, there was greater color degradation in mortars with 3% of TiO_2_ addition.

### 3.4. Compressive Strength

**Figure 10:**
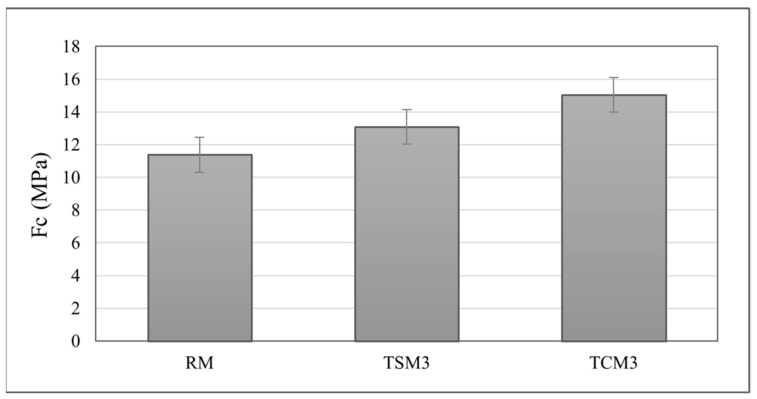
Compressive strength of RM, TCM and TSM mortar coatings (3% TiO_2_ addition by weight of cement).

Since the TSM3 and TCM3 mortars presented the highest photocatalytic activity in the degradation of dyes when exposed to UV radiation and visible light for 24 h with an additional increase in their activity over time and, on the other hand, also presented the greatest self-cleaning effect of the surfaces exposed to the weather conditions for the period of two months, showing cleaner surfaces, it was determined that the addition of nanoparticles of TiO_2_ at 3% by weight of cement was the optimal addition. Therefore, the mechanical test was only performed on these mortars to assess and verify that the compressive strength was not compromised. The particle size distribution of the fine limestone aggregates met the permissible particle size limits and an adjusted fineness modulus of 2.90 was calculated. The results of the compressive strength test are presented in the graph in Figure 10. After 28 days of curing, the compressive strength of the mortars with the addition of synthesized TiO_2_ increased by 14% when compared to the reference mortars, and for mortars with commercial TiO_2_, this resistance increased by 30%. All mortar mixtures with TiO_2_ addition experienced a decrease in fluidity and workability. Mortars made with both synthesized and commercial TiO_2_ addition required more water to reach the percentage of fluidity of the reference mortars. However, even with the water setting for the TCM and TSM mortars, compressive strength was not compromised. On the contrary, the compressive strength maintained a positive effect. According to the literature [39,40], TiO_2_ nanoparticles are an inert material and therefore they do not react with the components of the cementitious matrix. However, due to their size, they can act as filler agents for filling or refining pores, thereby reducing pore size and porosity; they may also act as nucleation sites, from which the cementitious paste hydration products grow, promoting the hydration process and material densification, which can be reflected in the increase in compressive strength [38,40,41,42].

In all comparative tests of the mortar samples with and without TiO_2_ additions, both commercial and synthesized, a similar behavior was found regarding the response to photocatalytic activity and the associated dye degradation efficiency. In decreasing order, from highest to lowest degradation efficiency, the following was found: TCM3 > TCM5 > TCM2 and TSM3 > TSM5 > TSM2. This pattern indicates that the mortar samples with 3% TiO_2_ nanoparticles added (both commercial and synthesized) presented the highest photocatalytic activity and the highest degradation efficiency. Although the mortars with a 5% addition of TiO_2_ nanoparticles contained a higher percentage of photocatalyst, the degradation efficiency remained below of that of the mortars with 3%, which may be attributed to a lower nanoparticle dispersion within the cementitious matrix, since by increasing the nanoparticle content, because of their tiny size, they tend to agglomerate and therefore decrease the active sites necessary for the photocatalysis reaction. Similar studies have been reported on photocatalytic cement-based mixtures with a high removal efficiency of organic contaminants; however, the high-temperature and high-relative-humidity weathering conditions significantly reduce the photocatalytic efficiency of TiO_2_ [18].

This research paper reports that the study was carried out particularly under real environmental conditions of high temperature and high relative humidity and, in addition to that, a limestone aggregate was used for the preparation of the cement-based mixtures (of high absorption); consequently, the generation and proliferation of micro-organisms on the cementitious surface were favored, promoting the biodegradation of the cementitious surfaces [43]. This research reports that the study was carried out particularly under real environmental conditions of high temperature and high relative humidity, in addition to that, limestone aggregate (high absorption) was used for the preparation of the base-cement mixtures; where, the generation and proliferation of micro-organisms is favored, promoting the biodegradation of cemented surfaces. Therefore, more research should be carried out for the real-scale application of photocatalytic building elements, which should be exposed to real weathering conditions.

## 4. Conclusions

In this study, anatase-TiO_2_ nanoparticles were synthesized at a low temperature (400 °C) by means of the sol–gel method. The results of the TiO_2_ characterization showed a crystallite size of 14.69 nm, a semi-spherical morphology and narrow particle size distribution, in the submicronic and nanometric order, which tends to increase the active surface area, inducing a greater degree of effectiveness as a photocatalytic agent. The photocatalytic activity of the synthesized TiO_2_ nanoparticles added in 2, 3 and 5% by weight of cement to coating mortars with limestone aggregates was determined, and compared to that of mortars with commercial TiO_2_ nanoparticles added and to mortars with no additions. The results of the study showed that mortars with 3% of TiO_2_ nanoparticles added, both synthesized and commercial, exhibited the highest photocatalytic activity, with a greater degradation efficiency for red wine and methyl orange dyes after exposure to both UV irradiation and visible light, when compared to mortars with additions of 5, 2% and no additions. Thus, a similar pattern was found with respect to photocatalytic activity and the associated dye degradation efficiency: in decreasing order, additions of 3, followed by 5 and 2%, for both synthesized and commercial TiO_2_, showed the best performances. The degradation efficiency of red wine for mortars with 3% commercial TiO_2_ was the highest when exposed to UV irradiation, while the degradation efficiency of red wine and methyl orange for mortars with the addition of 3% synthesized TiO_2_ was the highest in the coating mortars exposed to visible light. This is attributed to the TiO_2_ synthesis conditions, suggesting that synthetized TiO_2_ nanoparticles have a lower band gap that the commercial TiO_2_ nanoparticles, since less energy was required to induce photocatalysis when they were exposed to visible light. As for the mechanical resistance, the tests showed that mortars with 3% of synthesized or commercial TiO_2_ increased in compressive strength, when compared to mortars with no additions. For the studied period, a clear self-cleaning effect was observed on the surfaces of the mortars with TiO_2_ nanoparticle additions when exposed to weather under tropical climate conditions (high temperature and relative humidity and saline environment), where large surfaces of coating mortars become greatly susceptible to environmental attacks.

## Data Availability

All data, models and code generated or used during the study appear in the submitted article.
